# Biological Diversity and Parasitological Peculiarities of Myxosporea (Cnidaria, Myxozoa) Infecting *Merluccius Merluccius* (Linnaeus, 1758) in the Sea of Marmara

**DOI:** 10.1007/s11686-026-01232-1

**Published:** 2026-03-24

**Authors:** Derya Yadak, Cem Tolga Gürkanli, Sevilay Okkay, Yılmaz Çİftçİ, Ahmet Özer

**Affiliations:** 1 Fatsa Faculty of Marine Sciences, Department of Fisheries Technology Engineering, Ordu University, 52400 Fatsa, Ordu Turkey; 2 Institute of Science, Ordu University, 52200 Ordu, Turkey; 3 Science and Arts Faculty, Department of Biology, Kocaeli University, 41001 Kocaeli, Turkey; 4 Faculty of Fisheries and Aquatic Sciences, Sinop University, 57000 Sinop, Turkey

**Keywords:** Pseudalataspora vanderlingeni, Ceratomyxa, Phylogeny, Merluccius merluccius, Sea of marmara, Biodiversity

## Abstract

**Purpose:**

This study aimed to investigate the biodiversity and parasitological peculiarities of myxozoan parasites infecting European hake, *Merluccius merluccius*, one of the most commercially important demersal fish species, in the Sea of Marmara, Türkiye.

**Methods:**

A total of 47 *M. merluccius* specimens were collected from the eastern coasts of the Sea of Marmara and examined for myxozoan infections. Morphological observations, morphometric measurements, and phylogenetic analyses based on 18 S rDNA sequences were conducted to identify the parasites.

**Results:**

Of the 47 fish specimens examined, 12 were infected by two distinct myxozoan species. One was identified as *Pseudalataspora vanderlingeni* based on morphology, morphometry, and molecular data. The second species, *Ceratomyxa* sp., which could be identified to the genus level based solely on spore morphology, was consistently found co-infecting fish hosts of the previously identified parasite species. Notably, its spore morphology differed from that of the six *Ceratomyxa* species previously recorded in Turkish marine waters.

**Conclusion:**

This study presents the second global record of *Pseudalataspora vanderlingeni* since its original description, representing the first report of both the species and the genus *Pseudalataspora* in Turkish waters. Additionally, a new *Ceratomyxa* taxon from the same region in Turkish waters was also documented. Together with the previous reports, the results in this study suggest that *P. vanderlingeni* is a common myxozoan parasite of *Merluccius* species along the southern Atlantic coasts of Africa, with its range extending into the Mediterranean Sea and surrounding waters. Additionally, this study also presents *Ceratomyxa* as one of the possible common myxozoan parasites of *Merluccius* species in the Mediterranean and adjacent seas.

**Supplementary Information:**

The online version contains supplementary material available at 10.1007/s11686-026-01232-1.

## Introduction

The European hake, *Merluccius merluccius* (L., 1758), is one of the most economically important and therefore, one of the most heavily exploited demersal fish species in the Mediterranean Sea and its adjacent waters, including the Sea of Marmara [1, 2]. It is predominantly carnivorous and is generally found on muddy bottoms, with a wide depth distribution ranging from the coastline to 1000 m [3, 4]. It has a wide distribution across the Northeast Atlantic, extending from Norway to the Gulf of Guinea, the Mediterranean, and the Black Sea [1].

It has frequently been reported to be infected by metazoan parasites, including helminths and copepods [5–7]. In addition, members of another metazoan taxon, Myxozoa (Cnidaria), have also been reported as parasites of European hake and the other species of the genus *Merluccius* Rafinesque, 1810 [8–12]. Myxozoans are microscopic parasites with an extremely reduced body size and structure [13]. Currently, the class Myxozoa comprises more than 2,600 species across 65 genera and 17 families [14, 15]. Several myxosporean species have so far been reported from various hake species (*Merluccius hubbsi*-Argentine hake, *M. capensis*-Cape hake, *M. paradoxus*, *M. australis*-Southern hake, *M. productus*-Pacific hake, and *M. gayi*-Chilean hake) across countries with Atlantic and Pacific coastlines, including Argentina, Uruguay, South Africa, Canada, the USA, and the Mediterranean coastlines of France and Spain. These species include *Kudoa rosenbuschi, K. thyrsites, K. paniformis, Myxoproteus meridianalis, Pseudalataspora vanderlingeni, Ceratomyxa globulifera*, and *Fabespora* sp. Of these species, *K. thyrsites* and *K. paniformis* have been reported as the causative agents of post-mortem myoliquefaction (also known as milky, soft, or jelly flesh) in their hake hosts, resulting in significant economic losses [5, 7, 9–12, 16–21]. Despite its economic importance and relatively wide distribution, there are only two records of myxosporean infection in European hake *M. merluccius*: *Ceratomyxa globulifera* from the Mediterranean coast of France [17], and an unidentified coelozoic myxosporean parasite from the north-west Mediterranean coast of Spain [7].

Current literature lacks information on the diversity of myxosporean species infecting *M. merluccius*. Thus, this study aimed to elucidate the first biological diversity and infection characteristics of myxosporean species infecting *M. merluccius* in the Sea of Marmara, which lies adjacent to the Eastern Mediterranean Sea, as previously reported.

## Materials and Methods

### Fish Sampling and Parasitological Examination

Between January and December 2023, a total of 47 European hake, *M. merluccius* specimens (*n:* 17 in winter, *n:* 30 in summer), measuring 25.2–29.9 cm in length, were collected off the eastern coasts of the Sea of Marmara, specifically near Yalova and İzmit Bay (40°44′06′′ N, 29°′16′′ E), by local fishermen. Gills, fins, skin, urinary bladder, kidney, gallbladder, liver, intestine, smooth muscles, and gonads were microscopically examined for the presence of myxozoan parasites using a light microscope (Olympus BX51) equipped with a digital camera (DP71). Measurements of spore and polar capsule length and width were based on 30 fresh spores. All measurements were presented as mean values and min–max values in parentheses. Prevalence values (%) were calculated according to the definition by Bush et al. [22], while density values were determined using the density scale (1 + , 2 + , 3 + , 4 +) reported for myxosporean infections by Gürkanlı et al. [23].

### Molecular Analyses

Species-level identification of the myxozoan morphotypes observed in the infected *M. merluccius* tissues was conducted using phylogenetic analyses based on nucleotide sequences of the small subunit of ribosomal DNA (18S rDNA hereafter). Two myxozoan specimens from each morphotype, representing different sampling seasons, were included in the molecular analyses. Total genomic DNA from myxozoa-infected tissues were extracted using the PureLink® Genomic DNA Mini Kit (USA), according to the manufacturer’s instructions. Extracted DNA was stored at − 20 °C before use. The PCR amplification of the 18S rDNA locus was performed using primers MyxospecF [24] and 18R [25]. For amplification, a 50 μl PCR reaction was prepared using genomic DNA (< 0.5 μg), 1.5 mM MgCl_2_ (Invitrogen), 1.25 U *Taq* polymerase (Invitrogen), 0.8 mM dNTP mix (Thermo Scientific), 1 × PCR buffer (Invitrogen), 0.4 pmol (final concentration) of each primer, and ddH_2_O (up to 50 μl). A Techne (TC-Plus, Staffordshire, UK) thermal cycler was used for the PCR amplification with the following conditions: an initial denaturation at 95 °C for 3 min, followed by 40 cycles of denaturation at 94 °C for 1 min, annealing at 51 °C (− 0.1 °C/Cyc) for 1 min, and extension at 72 °C for 2 min. The procedure was completed with a final extension at 72 °C for 10 min. Subsequently, the PCR products were electrophoresed on a 1% agarose gel (prepared in 1X TBE buffer) and visualized using a Vilber Lourmat photo-print imaging system (France). Nucleotide sequencing was performed commercially by Macrogen-Europe (Amsterdam, the Netherlands) on both strands, using the same primers as those employed for the PCR amplifications. Assemblage and editing of the nucleotide sequences from both strands were conducted using BioEdit v.7.0.5.3 [26]. For phylogenetic analyses, a dataset was assembled from BLAST (Basic Local Alignment Search Tool; https://blast.ncbi.nlm.nih.gov/Blast.cgi) search results and the available literature (as shown in the table provided in the Online Resource). Multiple nucleotide sequence alignment of the data set was performed using MAFFT v.7 [27, 28]. Software BioEdit v.7.0.5.3 and Gblocks v.0.91b [29] were used to edit the alignment file. Akaike information criterion [30] and Bayesian information criterion tests were performed using the jModelTest v. 0.1 [31] software package to determine the best-fitting evolutionary model(s) for the data set. Phylogenetic constructions were conducted using Maximum-Likelihood (ML), Neighbor-Joining (NJ), and Maximum-Parsimony (MP) algorithms. Software PhyML 3.0 [32]was used for the ML analyses, whereas PAUP* v. 4.0b10 [33] was employed for the NJ and MP analyses. A heuristic search approach with a TBR swapping algorithm (10 random repetitions) was implemented for the MP analysis. The reliability of the phylogenetic relationships were assessed using Bootstrap tests performed with 1000 replicates. The new 18S rDNA genotypes obtained in this study have been deposited in GenBank under accession numbers PX856895- PX856896.

## Results

### Morphology and Light Microscopy

**Fig. 1:**
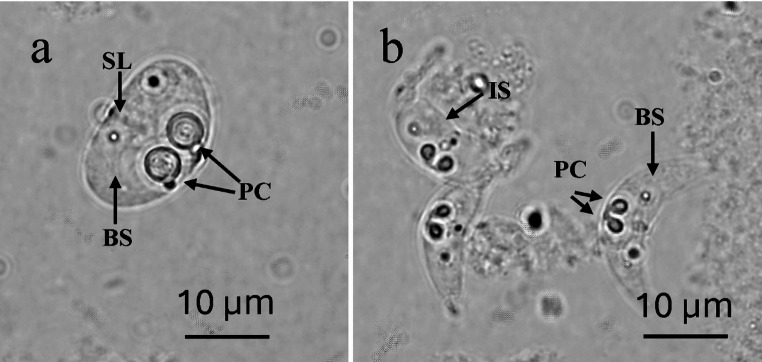
Spore morphology of myxosporeans (**a**) A fresh *Pseudalataspora* sp.-1 spore in valvular view, (**b**) Fresh *Ceratomyxa* sp.-1 spores in valvular view (PC: polar capsule; BS: binucleate sporoplasm; SL: suture line, IS: Immature spore)

Microscopic investigations of 12 infected fish hosts revealed two distinct myxospore morphotypes corresponding with the genera *Pseudalataspora* (Fig. 1a) and *Ceratomyxa* (Fig. 1b). The spore walls of all *Pseudalataspora* specimens observed consisted of two valve structures fused into an oval shape, with clearly visible and symmetrical margins. The valves exhibited a uniform, round-oval thickness and enclosed the binucleate sporoplasm (BS) and polar capsules (PC) evenly, indicating a symmetrical and regular valve architecture. Two polar capsules (PC) were oval with rounded ends, relatively large with a slight distance to one another, and were distinctly located near the center of the spore. They were positioned separately. The suture line (SL) between the valves was clearly defined, and two nuclei were present within the sporoplasm (Fig. 1a). The spores were measured, 14.0 μm (13.0–15.0 μm) in length, 19.6 μm (19.0–20.0 μm) in thickness, polar capsules 4.6 μm (4.0–5.0 μm) in length and 4.3 μm (4.0–5.0 μm) in width, polar capsule length: spore length = 1: 3.0–3.2; spore length: spore width = 1: 1.3–1.5. (Table 1). Due to similar spore morphologies and very close morphometric measurements, all Pseudalataspora specimens in this study were considered to belong to the same species, *Pseudalataspora* sp.-1.

**Table 1:** Site of infection, hosts, geographical localities, and dimensions (μm) of species of the genus *Pseudalataspora* and *Ceratomyxa* found in some marine fish (ND = not determined)

Species	Spore Body		Polar capsule		Site of infection	Host species	Locality	References
	Length	Thickness	Length	Width				
*Pseudalataspora* sp.-1	14.0 (13.0–15.0)	19.6 (19.0–20.0)	4.6 (4.0–5.0)	4.3 (4.0–5.0)	Gall bladder	*Merluccius merluccius*	Türkiye, Sea of Marmara	This study
*Pseudalataspora vanderlingeni*	14.2 ± 1.0 (12.2–15.3)	20.6 ± 1.8 (18.2–23.4)	5.0 ± 0.3 (4.4–5.5)	4.2 ± 0.3 (4.8–0.3)	Gall bladder	*Merluccius capensis*	South Africa	[12]
*Ceratomyxa* sp.-1	6.7 (6.0–7.0)	25.0 (22.0–28.0)	2.0 (1.9–2.0)	1.2 (1.0–1.5)	Gall bladder	*Merluccius merluccius*	Türkiye, Sea of Marmara	This study
*Ceratomyxa globulifera*	10.0	50.0	ND	ND	Gall bladder	*Merluccius vulgaris*	France, Mediterranean Sea	[17]
*C. globulifera*	8.0–10.0	52.0–58.0	4.0–5.0	ND	Gall bladder	*Ophidion rochei*	Russia, Black Sea	[34]
*C. scophthalmi*	9.4 (8.5–10.0)	49.6 (44.5–55.0)	3.1 (2.7–3.5)	3.2 (2.7–3.5)	Gall bladder	*Scophthalmius maeoticus*	Türkiye, Black Sea	[35]
*C. elegans*	6.0–9.1	20.0–35.5	2.4–2.5	2.2–2.4	Gall bladder	*Scorpaena porcus*	Ukraine, Black Sea	[36]
*C. hepseti*	6.0–8.0	10.0–15.0	6.0–8.0		Gall bladder	*Atherina hepsetus*	Ukraine, Black Sea	[37]
*C. inaequalis*	5.0 (5.0–6.0)	28.0 (28.0–31.0)	2.5–3.5	ND	Gall bladder	*Sypmhodus* spp.	Ukraine, Black Sea	[38]
*C. markewichi*	4.8–6.0	16.0–20.4	2.0–2.5	1.3–1.8	Gall bladder	*Trachurus mediterraneus*	Ukraine, Black Sea	[39]
*C. merlangi*	5.5 (5.0–5.8)	32.2 (27.6–34.8)	2.7 (2.4–2.9)	2.2 (1.9–2.3)	Gall bladder	*Merlangius merlangus*	Türkiye, Black Sea	[40]
*C. peculiaria*	6.5–8.5	21.0–29.3	2.4–2.7	1.9–2.4	Gall bladder	*Spicara flexuosa*	Ukraine, Black Sea	[41]
*C. allantoidea*	6.0–6.6	29.3–38.6	2.0–2.7	1.0–1.5	Gall bladder	*Ammodytes tobianus*	Bay of Biscay	[42]
*C. leatherjacketi*	5.8–6.5	24.5–31.0	2.5–3.0		Gall bladder	*Aluterus monoceros*	West coast of Malaysia	[43]

The second myxosporean species observed in this study was assigned to the genus *Ceratomyxa* based on its characteristic features, including two-valved, arcuate spores. Additionally, a distinctive symmetry was detected in the valves tapering to rounded tips. Moreover, sporoplasm binucleate (BS), polar capsules (PC) were identified as slightly pear-shaped toward the ends and located centrally within the spore (Fig. 1b). Developing immature spores (IS), often ‘crumpled’ in shape were observed (Fig. 1b). The measurements from 30 spore individuals revealed the following results: spore length was 6.7 μm (6.0–7.0 μm), spore thickness was 25.0 μm (22.0–28.0 μm), polar capsule length was 2.0 μm (1.9–2.0 μm), and polar capsule width was 1.2 μm (1.0–1.5 μm) (Table 1). Based solely on the significant similarities in spore morphology and morphometric measurements, all *Ceratomyxa* specimens in this study were classified as a distinct morphotype, *Ceratomyxa* sp.-1.

### Phylogenetic Investigations

Specimens AO-93 and AO-95 were included in the molecular analyses as representatives of *Pseudalataspora* sp.-1. However, molecular analyses could not be conducted for *Ceratomyxa* sp.-1, because all specimens of this species were found mixed with *Pseudalataspora* sp.-1.

**Fig. 2:**
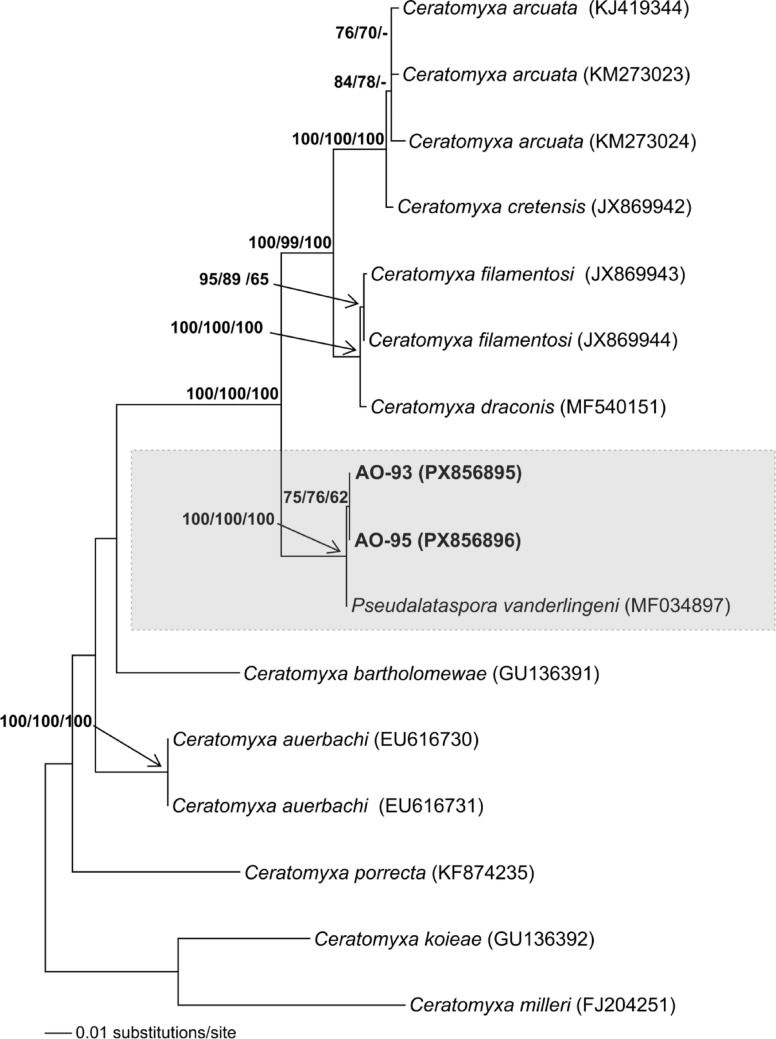
The NJ tree, based on 18S rDNA nucleotide sequences of *Pseudalataspora* sp.-1 genotypes obtained in this study (AO-93 and AO-95) and closely related myxozoan species obtained from NCBI (GenBank accession numbers are indicated on the tree. Additional details are provided in the Online Resource). The tree is created using the GTR + I + G model, and Bootstrap values (≥ 50%) from NJ, ML, and MP analyses are displayed in the respective order at each relevant node. The tree is rooted with *Ceratomyxa koieae* and* C. milleri*

The 18S rDNA nucleotide sequences obtained from the selected *Pseudalataspora* sp.-1 specimen, AO-93 and AO-95, were approximately 1435 bp in length. The nucleotide sequence similarity between these two genotypes was 99.7%. The BLAST search identified both of these genotypes as the closest to *Pseudalataspora vanderlingeni* Reed, Kalavati, Mackenzie, Collins & Hemmingsen, 2018. Accordingly, a dataset comprising the related myxozoan species was established (see Online Resource). Phylogenetic analyses were performed on 1,165 aligned nucleotides, including 239 variable sites and 324 mutations. The GTR + I + G (I: 0.582; G: 0.617) and TIM3ef + I + G (I: 0.590; G: 0.642) evolutionary models were suggested by the AIC and BIC tests, respectively. The ML and NJ trees, derived from the initial model, were preferred because they had higher bootstrap support. The MP analysis, conducted using 162 synapomorphic sites, produced a single most parsimonious tree with 422 steps (CI: 0.767773; RI: 0.827768 and HI: 0.232227). In all phylogenetic trees constructed using the NJ, ML, and MP algorithms, specimens AO-93 and AO-95 appeared as sister taxa, with P. vanderlingeni (MF034897) as the closest binomial species to them. The nucleotide sequence identities of AO-93 and AO-95 with *P. vanderlingeni* were 99.5% and 99.7%, respectively. Additionally, the relationships within this lineage, comprising these three specimens, were supported by significant bootstrap values (62% ≤) in all phylogenetic trees (Fig. 2).

### Parasitological Investigations

Of the 47 European hake (M. merluccius) samples investigated, 12 (25.5%) were infected with two myxosporean species. belonging to the genus *Pseudalataspora* Kovaljova & Gayevskaya, 1983 (*Pseudoalataspora* sp-1) and *Ceratomyxa* Thélohan, 1892 (*Ceratomyxa* sp-1) occurring either as sole species or co-infections in the bile of the gall bladder in the infected hosts.

*Pseudoalataspora* sp.-1 individuals were determined in all of the infected host fishes (*n:* 12), either as the sole parasite (*n:* 7) or co-occurring with *Ceratomyxa* sp.-1 spores (*n:* 5). The overall prevalence of infection and density of *Pseudoalataspora* sp.-1 were calculated as 25.5% and 3 + , respectively. Seasonal prevalence and infection density were also determined and are presented in Table 2. *Ceratomyxa* sp.-1, the second myxozoan species identified in this study, was found in 5 out of 47 host fishes, corresponding to a prevalence of 10.6%, together with an infection density of 3 + . Seasonal prevalence and density values of infection of *Ceratomyxa* sp.-1 were also determined and provided in Table 2. It is worth noting that *Ceratomyxa* sp.-1 was observed exclusively co-occurring with *Pseudoalataspora* sp.-1 rather than as a sole parasite species in the bile of host fishes.

**Table 2:** Overall and seasonal levels of infection prevalence (%) and density of *Pseudalataspora vanderlingeni*, and co-existing *P. vanderlingeni* + *Ceratomyxa* sp. in European hake, *M. merluccius*.N = number of fish examined

Infection indices	Season	Parasite	
		*Pseudalataspora vanderlingeni*	Co-infection (*P. vanderlingeni* + *Ceratomyxa* sp.)
Prevalence (%)	Winter (n = 17)	29.4	11.8
	Summer (n = 30)	23.3	10.0
	Overall (n = 47)	25.5	10.6
Density	Winter (n = 17)	3 +	3 +
	Summer (n = 30)	4 +	3 +
	Overall (n = 47)	3 +	3 +

## Discussion

This study presents valuable new information on the biodiversity and parasitological characteristics of Myxosporea infecting *M. merluccius* in the Sea of Marmara, Türkiye. Both microscopic and phylogenetic examinations revealed that two distinct spore types, belonging to the genera *Pseudalataspora* and *Ceratomyxa*, were present in the gallbladders of infected host fishes. Morphological and morphometric data, as well as phylogenetic analyses of the 18S rDNA gene with a high bootstrap value (100%) for *Pseudalataspora* sp.-1 spores, revealed that all samples belonged to the same species, *Pseudalataspora vanderlingeni* Reed, Kalavati, Mackenzie, Collins & Hemmingsen, 2018 (MF034897).

### Morphology and Light Microscopy

When comparing the spore morphology and morphometric data of *Pseudalataspora* sp.-1 with the original description of *P. vanderlingeni* [12], it is evident that the two species are almost indistinguishable (see Table 1 for details), with only slight differences. The original description noted relatively small alate processes that joined at the proximal extremities of the valves; these were observed only in some developing spores in the present study. In the present study, the shape and dimensions of polar capsules were mostly round, with a uniform margin, whereas in the original spores, they were oval, with varying dimensions and margins. As a result, all evidence obtained from spore morphology, morphometry, and molecular phylogeny led us to assign *Pseudalataspora* sp.-1 spores to *Pseudalataspora vanderlingeni*. This species is relatively new, having been observed and identified in the gallbladder of the type host, the Cape hake, *Merluccius capensis* Castelnau, 1861, in South Africa. Additionally, *M. paradoxus* Franca, 1960, from the west coast of Africa, has been reported as another host of this parasite [12]. Since it’s the first identification, there have been no subsequent records of this parasite. Therefore, this study presents the second global record of *P. vanderlingeni*. So far, several myxozoan genera, including *Myxobolus*, *Henneguya*, *Ortholinea*, *Kudoa*, *Ceratomyxa,* and *Myxidium,* have been reported in various fish species along the Turkish coasts [23, 35, 40, 44–54]. However, although a single species, *Pseudalataspora pontica* Kovaljova, Donec & Kolesnikova, 1989 (ext. from *Chelon aurata*), has been identified in the northern Black Sea, there has been no previous record of infection by members of the genus *Pseudalataspora* in Türkiye. Therefore, this study provides the first record of both the species *P. vanderlingeni* and the genus *Pseudalataspora* in Turkish waters.

The second myxozoan genus identified in this study was *Ceratomyxa*, which was observed only in co-infection with *P. vanderlingeni* spores. The defining characteristics of the genus are crescent-shaped or arcuate spores and elongated shell valves that are generally thicker than they are long [55]. Homogeneity in morphometric measurements, coupled with the presence of characteristically arcuate and equal spore valves, indicated that all *Ceratomyxa* specimens in this study belong to the same morphotype: *Ceratomyxa* sp.-1. However, molecular data could not be obtained from these specimens to enable a precise species identification. To date, six binomial species of the *Ceratomyxa* and two unspecified specimens of the genus have been recorded from marine fishes in the Turkish seas. These include: *Ceratomyxa* sp. (extracted from *Dicentrarchus labrax*), *Ceratomyxa beloneae* (ex. from *Belone belone*), *C. merlangi* (ex. from *Merlangius merlangus*), *Ceratomyxa* sp. (ex. from *Scophthalmus maeoticus*), *C. scorpaeni* (ex. from *Scorpaena porcus*), *C. scophthalmi* (ex. from *S. maeoticus*), *C. diplodae* and *C. labracis* (ex. from *D. labrax*) [35, 48, 50–54]. Although all these species exhibited equal-valved spores, *Ceratomyxa* sp.-1 exhibited morphometric characteristics that did not correspond exactly to any previously recorded species in the Black Sea or other regions (see Table 1 for details), suggesting that it is likely a new taxon from Turkish waters. Additionally, when compared to other morphologically similar and equally valved *Ceratomyxa* species distributed worldwide (as reported by Eiras [56] and Eiras et al. [57], as well as by several authors in the Black Sea (see Table 1 for details), some species exhibited mostly overlapping spore and polar capsule dimensions, but different spore morphology. These species include *C. merlangi*, which has crescent-shaped spores with blunt, rounded ends very closely located polar capsules to each other; *C. peculiaria*, which has valves that are totally round-ended with an anterior margin that is slightly convex and a posterior margin that is straight or slightly convex; and *C. leatherjacketi*, which has spores that are crescent-shaped and transversely elongated with a posterior angle that is slightly concave, as well as valves that are equal in size or one that is slightly elongated, and spherical polar capsules. Some of the "crumpled" spores observed in the present study were also previously reported for *C. scophthalmi* [35]. It must be noted that the traditional taxonomic classification based on spore and polar capsule morphology may not be sufficient for a proper species identification because several myxosporeans with very similar spore morphology assigned to the same genera have been located in phylogenetically distantly related taxa. Given the lack of genetic data in the present study and the morphological differences among the above-mentioned species, our *Ceratomyxa* sp.-1 remains a distinct morphotype of a likely new taxon based on current data; however, further studies will be required to confirm the exact species identification.

### Phylogenetic Investigations

The genus *Pseudalataspora* comprises 14 species; however, only two, *P. kovalevae* and *P. vanderlingeni*, have available genetic data. Phylogenetically, members of this genus have been found in different lineages within the *Ceratomyxa* clade, forming a paraphyletic group [12, 58, 59]. Therefore, the boundaries of genetic variation established within and between *Ceratomyxa* species are also applicable to *Pseudoalataspora* species. A low intraspecific 18S rDNA variation has been reported for the genus *Ceratomyxa*. For instance, this value was 0.15% (99.85% similarity) in *C. nolani* and 0.36% (99.64% similarity) in *C. lunula* [55]. Additionally, the genus has been characterised by high interspecific divergence in the 18S rDNA gene. Reported genetic similarities among closely related species include 98.5% between *C. sultani* and *C. arabica*, 97.6% between *C. gunterae* and *C. dennisi*, and 97.6% between *C. robertsthomsoni* and *C. thalassomae* [35, 55]. From this perspective, the high nucleotide sequence similarity (99.7% and 99.5%) between *Pseudalataspora* sp.-1 (AO-93 and AO-95) and *P. vanderlingeni* specimens supported the conclusion that these two taxa belonged to the same species.

### Parasitological Investigations

All species of the genus *Pseudalataspora*, including *P. vanderlingeni*, are coelozoic parasites that have been found in the gall bladders of marine fishes [12]. In line with the original description of *P. vanderlingeni*, all specimens examined in this study were observed in the gall bladder of the host fish, *M. merluccius*. Consequently, the *P. vanderlingeni* spores examined in this study occupy the same ecological niche as those originally described. Similarly, the genus *Ceratomyxa* comprises more than 270 species that predominantly inhabit the gallbladder [57]. Consistent with this background, all *Ceratomyxa* sp.-1 specimens in this study were observed in the gall bladder of infected *M. merluccius* individuals.

The prevalence of *P. vanderlingeni* infections in the two originally reported host species, *M. capensis* and *M. paradoxus*, collected from the Southern Benguela off the West Coast of South Africa, was 100%. By contrast, the prevalence was 61.5% in *M. capensis* in the southern Benguela region [12]. The overall prevalence of infection in this study (25.5%) is lower than that reported in the aforementioned studies, but higher than the prevalence of *P. misrae* (3.8%) in Rastrelliger kanagurta [60] and within the range reported for *P. lophii* in *Lophius piscatorius* [35] and *P. beryxi* in *Beryx splendens* [61]. The overall prevalence of co-occurring *P. vanderlingeni* and *Ceratomyxa* sp. infections in the present study was 10.6%, which is double the reported prevalence (4.76%) of co-occurring *P. lophii* and *C. lophii* infections in *L. piscatorius* [35]. Despite limited data on infections involving members of the genus *Pseudalataspora*, there are more reports of single and co-infections involving members of the genus *Ceratomyxa* in fish hosts, with overall prevalence rates of up to 100% and marked seasonal variation [35, 53, 57, 62–65]. Myxosporean co-infections have been linked to biological, geographical, and environmental factors, as well as to similar life cycle strategies [53, 66]. Our results revealed very similar overall and seasonal (winter and summer) infection prevalence values for *P. vanderlingeni* and its co-infections with *Ceratomyxa* sp.-1. A two-stage life cycle strategy involving a myxosporean stage in fish and an actinosporean stage in invertebrate alternate hosts in freshwater environments, and fish-to-fish transmission in marine myxosporean parasites, has been reported [67–73]. Alama-Bermejo et al. [63] reported the presence of the invasive blood stage of *C. puntazzo* in sharpsnout seabream (*Diplodus puntazzo*) year-round, as determined by PCR. Based on their unsuccessful experimental transmission of different *C. puntazzo* developmental stages in seawater or via oral and intracoelomic injection, they hypothesized that an invertebrate host was involved in life-cycle dynamics rather than fish-to-fish transmission in aquaculture systems. Currently, no reports indicate how infections with *P. vanderlingeni* and *Ceratomyxa sp*.-1 progress. Based on the aforementioned reports on the life-cycle strategies of marine myxosporean parasites and the infection levels of the two parasite species identified in our study, it can be inferred that they likely share the same life-cycle strategy: either an alternate or a direct fish-to-fish transmission. According to Rocha et al. [74], temperature is the primary factor influencing the life cycle of myxosporean parasites, facilitating their successful development alongside specific physical, chemical, and biological conditions. The Benguela system, the locality where the original description of *P. vanderlingeni* was reported, is one of the four major eastern boundary upwellings of the World Ocean and is unique because its warm water currents with enhanced plankton production, which are requested by pelagic and demersal resources, including fish, for food [75, 76]. On the other hand, the biodiversity in the Sea of Marmara, where this study was conducted, is threatened by low dissolved oxygen levels, increasing eutrophication caused by human impacts, and marine litter on the seafloor.[77]. The differences in the overall infection prevalence values of *P. vanderlingeni* in the original description (61,5–100%) and in the present study (25.5%) could have resulted from these different characteristics of both sampling localities. Alama-Bermejo et al. [63] also reported temperature-related seasonal *C. puntazzo* infections in fish, coinciding with temperature values, and indicated that temperature appeared to regulate the density of actinospores of *C. puntazzi* in the environment in accordance with their hypothesized invertebrate–fish host life cycle strategy. Similar seasonal patterns observed for both parasite species in the present study may also be due to these factors being available year-round to the parasites or their alternate hosts.

Various myxozoan genera, including *Kudoa, Myxoproteus, Fabespora, Pseudalataspora,* and *Ceratomyxa,* have been reported to infect *Merluccius* species, some of which have either a widespread or a restricted global distribution. Of these, *C. globulifera* has only ever been found in *M. merluccius* off the Mediterranean coast of France, and *P. vanderlingeni* has only ever been found once in *M. capensis* and *M. paradoxus* along the west and south coasts of South Africa. However, the results of this study suggest that *P. vanderlingeni* and *Ceratomyxa* have a broader distribution, extending into the eastern Mediterranean Sea and neighbouring seas. This indicates that *P. vanderlingeni* could be a widespread parasite of *Merluccius* species, affecting populations not only off the African coast, but also in the Mediterranean Sea. Similarly, *Ceratomyxa* appears to be prevalent in *Merluccius* populations throughout the Mediterranean Sea. Nevertheless, further studies on myxosporean occurrences in a wider range of host species and geographical locations are required to confirm these presumptions.

The main findings of this study can be summarised as follows; (a) Members of two myxozoan taxa (*P. vanderlingeni* and *Ceratomyxa* sp.-1) were identified in *M. merluccius* caught in the Sea of Marmara in Türkiye; (b) This study presents the second global record of *P. vanderlingeni* since its original description, and the first record of the genus *Pseudalataspora* and *P. vanderlingeni* in Turkish waters; (c) Based on unique spore morphology, this study presents a likely new *Ceratomyxa* taxon in Turkish waters; (d) The results of this study, in conjunction with those of Reed et al*.* [12], suggest that *P. vanderlingeni* is a common myxozoan parasite of *Merluccius* species along the Southern Atlantic coasts of Africa, extending into the Mediterranean and surrounding seas; (e) Similarly, in conjunction with the results of Desportes and Theodorides [17], this study presents *Ceratomyxa* is one of the common myxozoan parasite of *Merluccius* species in the Mediterranean and adjacent waters.

## Supplementary Information

Below is the link to the electronic supplementary material.

Supplementary Material 1

Supplementary Material 2

## Data Availability

Sequence data is available on the NCBI GenBank database. All other necessary data are included in the article.
