# Supplementary material for: Biological Diversity and Parasitological Peculiarities of Myxosporea (Cnidaria, Myxozoa) Infecting Merluccius Merluccius (Linnaeus, 1758) in the Sea of Marmara
Source: Acta Parasitol. 2026 Mar 24;71(2):73. doi: 10.1007/s11686-026-01232-1 (PMC13013176; doi:10.1007/s11686-026-01232-1)
Supplement: Supplementary file 1 — Supplementary Material 1 [file 11686_2026_1232_MOESM1_ESM.docx]

**Biological Diversity and Parasitological Peculiarities of Myxosporea (Cnidaria, Myxozoa) Infecting *Merluccius merluccius* (Linnaeus, 1758) in the Sea of Marmara**

Derya YADAK^1,2^, Cem Tolga GÜRKANLI^1^*, Sevilay OKKAY^3^, Yılmaz ÇİFTÇİ^1^ & Ahmet ÖZER^4^

*^1^Ordu University, Fatsa Faculty of Marine Sciences, Department of Fisheries Technology Engineering, 52400, Fatsa-Ordu/Türkiye*

*^2^Ordu University, Institute of Science, 52200, Ordu/Türkiye*

^3^*Kocaeli University, Science and Arts Faculty, Department of Biology, 41001, Kocaeli, Türkiye*

^4^*Sinop University, Faculty of Fisheries and Aquatic Sciences, 57000, Sinop, Türkiye*

*Corresponding author: [ctgurkanli@odu.edu.tr](mailto:ctgurkanli@odu.edu.tr) / cgurkanli44@gmail.com; <https://orcid.org/0000-0001-8378-7109>

**Online Resource**: Source information of *Pseudalataspora* sp.-1 (*P. vanderlingeni*) genotypes obtained in this study, in addition to 18S rDNA genotypes of closely related *Myxozoa* species obtained from GenBank for phylogenetic analyses

| **Species** | **Host** | **Locality** | **Tissue/Organ** | **Acc. No.** | **Source** |
| --- | --- | --- | --- | --- | --- |
| **AO-93** | *Merluccius merluccius* | Türkiye | Bile | PX856895 | This study |
| **AO-95** | *Merluccius merluccius* | Türkiye | Bile | PX856896 | This study |
| *Pseudalataspora vanderlingeni* | *Merluccius capensis* | South Africa | Gall bladder | MF034897 | [12] |
| *Ceratomyxa arcuata* | *Lophinus piscatorius* | Scotland | Gall bladder | KJ419344 | [78] |
| *Ceratomyxa arcuata* | *Lophinus piscatorius* | Scotland | Gall bladder | KM273023 | [78] |
| *Ceratomyxa arcuata* | *Collionmus lyra* | Scotland | Gall bladder | KM273024 | [78] |
| *Ceratomyxa cretensis* | *Synodus saurus* | Greece | Bile | JX869942 | [79] |
| *Ceratomyxa filamentosi* | *Synodus saurus* | Greece | Bile | JX869943 | [79] |
| *Ceratomyxa cretensis* | *Synodus saurus* | Greece | Bile | JX869944 | [79] |
| *Ceratomyxa draconis* | *Trachinus draco* | Tunisia | Gall bladder | MF540151 | [80] |
| *Ceratomyxa bartholomewae* | *Hyporhamphus dussumicri* | Australia | Gall bladder | GU136391 | [81] |
| *Ceratomyxa auerbachi* | *Clupea harengus* | Denmark | Gall bladder | EU616730 | [61] |
| *Ceratomyxa auerbachi* | *Clupea harengus* | Denmark | Gall bladder | EU616731 | [61] |
| *Ceratomyxa porrecta* | *Myoxocephalus scorpus* | Norway | Gall bladder | KF874235 | [82] |
| *Ceratomyxa koieae* | *Sphyraena forsteri* | Australia | Gall bladder | GU136392 | [81] |
| *Ceratomyxa milleri* | *Lutjanus fulviflamma* | Australia | Gall bladder | FJ204251 | [47] |

1. Kalatzis PG, Kokkari C, Katharios P (2013) Description and relationships of two novel species of *Ceratomyxa* Thelohan, 1892 infecting the gallbladders of *Aulopiformes*: Atlantic lizardfish *Synodus saurus* Linnaeus, 1758 and royal flagfin *Aulopus filamentosus* Bloch, 1792 from the Cretan Sea, Greece. Parasitol Res 112:2055-2061. https://doi.org/10.1007/s00436-013-3366-7
2. Azizi R, Yemmen C, Rangel LF, Santos MJ, Bahri S (2020) Morphology, seasonality and molecular characterization of *Ceratomyxa draconis* n. sp. parasite of *Trachinus draco* (L.) from the Bay of Bizerte, Tunisia. Parasitol Res 119:2431-2438. https://doi.org/10.1007/s00436-020-06664-w
3. Gunter NL, Burger MAA, Adlard, RD (2010) Morphometric and molecular characterisation of four new *Ceratomyxa* species (Myxosporea: Bivalvulida: *Ceratomyxidae*) from fishes off Lizard Island, Australia. Folia Parasitol 57:1-10. <https://doi.org/10.14411/fp.2010.001>
4. Kodádková A, Dyková I, Tyml T, Ditrich O, Fiala I (2014) Myxozoa in the High Arctic: Survey on the central part of Svalbard archipelago. Internat J Parasitol: PAW, 26 3:41-56. https://doi.org/10.1016/j.ijppaw.2014.02.001
